# Direct Inhibition of Microglia Activation by Pretreatment With Botulinum Neurotoxin A for the Prevention of Neuropathic Pain

**DOI:** 10.3389/fnins.2021.760403

**Published:** 2021-12-07

**Authors:** Xiaona Feng, Donglin Xiong, Jie Li, Lizu Xiao, Weijiao Xie, Yunhai Qiu

**Affiliations:** ^1^Research Center for Neural Engineering, Shenzhen Institutes of Advanced Technology, Chinese Academy of Sciences, Shenzhen, China; ^2^School of Medicine, Southern University of Science and Technology, Shenzhen, China; ^3^Shenzhen Municipal Key Laboratory for Pain Medicine, Department of Pain Medicine, Shenzhen Nanshan People’s Hospital, The 6th Affiliated Hospital of Shenzhen University Health Science Center, Shenzhen, China; ^4^Department of Anesthesiology, Shenzhen Second People’s Hospital, Shenzhen, China

**Keywords:** botulinum neurotoxin A (BoNT/A), neuropathic pain, microglia activation, pretreatment, BoNT/A intrathecal administration

## Abstract

Peripheral injection of botulinum neurotoxin A (BoNT/A) has been demonstrated to have a long-term analgesic effect in treating neuropathic pain. Around peripheral nerves, BoNT/A is taken up by primary afferent neurons and inhibits neuropeptide release. Moreover, BoNT/A could also be retrogradely transported to the spinal cord. Recent studies have suggested that BoNT/A could attenuates neuropathic pain by inhibiting the activation of spinal glial cells. However, it remains unclear whether BoNT/A directly interacts with these glial cells or via their interaction with neurons. Our aim here is to determine the direct effect of BoNT/A on primary microglia and astrocytes. We show that BoNT/A pretreatment significantly inhibits lipopolysaccharide (LPS) -induced activation and pro-inflammatory cytokine release in primary microglia (1 U/mL BoNT/A in medium), while it has no effect on the activation of astrocytes (2 U/mL BoNT/A in medium). Moreover, a single intrathecal pre-administration of a low dose of BoNT/A (1 U/kg) significantly prohibited the partial sciatic nerve ligation (PSNL)- induced upregulation of pro-inflammatory cytokines in both the spinal cord dorsal horn and dorsal root ganglions (DRGs), which in turn prevented the PSNL-induced mechanical allodynia and thermal hyperalgesia. In conclusion, our results indicate that BoNT/A pretreatment prevents PSNL-induced neuropathic pain by direct inhibition of spinal microglia activation.

## Introduction

Neuropathic pain (NP) results from dysfunctions in the sensory nervous system response due to pathology. The development of NP involves aberrant excitability of the nervous system in primary sensory ganglia (peripheral sensation) and the spinal cord dorsal horn (central sensitization). Beyond neurons, spinal microglia and astrocytes play critical roles in central sensitization. Spinal microglia are activated as early as 1 day after peripheral nerve injury and make a critical contribution to the initiation and maintenance of pathological enhanced pain hypersensitivity (Romero-Sandoval et al., 2008; Inoue and Tsuda, 2009). Neuropathic pain patients suffer serious symptoms, including spontaneous pain, hyperalgesia and allodynia, which dramatically decreases their quality of life. Currently, however, available drugs for treatments are limited and there is a lack of effective therapy.

Botulinum neurotoxin A (BoNT/A) is a globally approved medication used for cosmetic and therapeutic treatment. In addition to the well-known action as a muscle relaxant, BoNT/A is approved for the treatment of pain in chronic migraine and cervical dystonia and, as an off-label therapy, in other painful pathologies including NP in humans (Matak et al., 2019). The effective analgesic effect of BoNT/A is due to the inhibition of neurotransmitters and neuropeptide release from neurons involved in nociceptive transmission. BoNT/A enters cytosol via vesicle endocytosis when binding to synaptic vesicle glycoprotein 2 (SV2) in nerve endings (Dong et al., 2006), preventing neurotransmitter release by cleaving synaptosomal-associated protein 25 (SNAP-25) (Schiavo et al., 1993; Padda and Tadi, 2021). Interestingly, BoNT/A shows long-term antinociceptive properties, and it is believed that the regulation of central sensitization is involved (Luvisetto, 2020). BoNT/A acts not only at the peripheral administration site, but is also retrogradely transported to dorsal root ganglion (DRG) and spinal dorsal horn sensory neurons (Habermann, 1974; Marinelli et al., 2012), thereby blocking the release of neurotransmitters from central endings of primary afferent neurons, resulting in reduced central sensitization (Cui et al., 2004; Park and Chung, 2018).

In addition to the action of BoNT/A on neurons, multiple studies have suggested that the analgesic effect of BoNT/A could also be mediated by spinal glial cells. Single intraplantar administration of BoNT/A has been demonstrated to not only attenuate neuropathic pain-related behaviors but also reduce the activation of microglia (Mika et al., 2011; Vacca et al., 2013; Zychowska et al., 2016) and the number of astrocytes in rat spinal cord dorsal horn (Vacca et al., 2013). However, it is generally believed that the effect of BoNT/A on spinal microglia and astrocytes could be indirect because BoNT/A is administrated in peripheral nerves, in which microglia and astrocytes are absent. Hence, it remains unclear whether BoNT/A can directly interact with microglia and astrocytes. To reveal whether BoNT/A could have a direct interaction with microglia or astrocytes, we pretreated cultured microglia or astrocytes with BoNT/A and checked for the LPS-induced activation of microglia and astrocytes when free BoNT/A was removed. We further intrathecally pre-injected BoNT/A into rats to inhibit the initial activation of microglia and checked the analgesic effects of partial sciatic nerve ligation (PSNL)-induced neuropathic pain-like behaviors.

## Materials and Methods

### Animals

Male Sprague-Dawley rats weighing 250–300 g at the time of surgery were sourced from the Guangdong Medical Lab Animal Center. Rats were house kept on a 12 h light/dark cycle at 24°C with free access to food and water at a SPF barrier of animals’ facility during the processes of surgical and behavior test. All husbandry and experimental procedures were approved by the Animal Care and Use Ethics Committees at Shenzhen Institutes of Advanced Technology, Chinese Academy of Sciences Research Committee of Laboratory Animals.

### Chemicals

Poly-L-lysine (PLL, P4707), dispase II (D4693) and collagenase type IV (C5138) were purchased from Sigma-Aldrich; Dulbecco’s Modified Eagle Medium (DMEM, 11995065), fetal bovine serum (FBS, 10099141) and Penicillin-Streptomycin (15070063) were purchased from Gibco; ReverTra Ace^®^ qPCR RT Kit (FSQ-101) and SYBR Green^®^ Realtime PCR Master Mix (QPS-201) were purchased from Toyobo; Total RNA extraction kit (EK02605) were purchased from Ensure Biologicals; BoNT/A (BOTOX, 100 U) was Allergan Pharmaceuticals Ltd., Inc.

### Cell Cultures

For primary microglia cultures, microglia were prepared from the cerebral cortices of newborn Sprague-Dawley rats via a previously reported method with minor modifications (Saura et al., 2003). Ten seconds after alcohol immersion, rat pups (postnatal day 1, *n* = 8–10) were decapitated. Cerebral cortex was then isolated, cut into small pieces and dissociated with 2.5 mg/mL dispase II and 0.5 mg/mL collagenase type IV for 30 min. Mixed cells were seeded in a PLL -coated six-well-plate at a density of 5 × 10^4^ in DMEM medium with 10% FBS and 50 U/ml Penicillin-Streptomycin. Mixed cells cultured at 37°C in a humidified 5% CO_2_/95% air and the culture medium were replaced every 3 days. After 20 days of culturing, the mixed cells were mildly trypsinized (0.025% trypsin in DMEM) at 37°C for 1 h. Floating cells were removed and rinsed once with pre-warmed DMEM medium. Purified microglia were cultured in DMEM containing 10% heat- inactivated FBS, penicillin and streptomycin. The purities (99%) of these cultures were confirmed by Iba1 immunostaining.

For primary astrocyte cultures, astrocytes were prepared from the cerebral cortices of newborn Sprague-Dawley rats as previously described (Menet et al., 2001). Mixed cells were obtained from the cerebral cortex of rat pups (postnatal day 1, *n* = 4–6) followed by mechanical dissociation, dispersion, centrifugation and cultured in a PLL-coated 75 cm^2^ flask at density of 5 × 104 cells (all these steps were identical to those described for mixed cells culture in primary microglia section above). Astrocytes were purified by shaking to remove the top layer miscellaneous cells. The purities (99%) of these cultures were confirmed by GFAP immunostaining.

### Immunostaining

Purified microglia or astrocyte cells were first washed three times by warmed PBS, fixed with pre-cooled 4% paraformaldehyde (PFA) for 10 min, and then washed by cold PBS for 10 min. After blocking for 1 h, cells were incubated with primary antibodies for 2 h at room temperature, followed by 3 washes with PBS. The corresponding secondary antibodies (1:300) containing DAPI solutions (1:1,000) were applied for another 1 h. After washed out, images were obtained with a fluorescence microscope (IX71, Olympus, PA). The following primary antibodies were used: goat anti-Iba1 (1:200; AF1039a, Abcepta) and chicken anti-GFAP (1:400; ab4674, Abcam). The following secondary antibodies were used: anti-chicken IgY H&L Alexa Fluor^®^ 647 (1:300, ab150171, Abcam) and anti-goat IgG H&L Alexa Fluor^®^ 488 (1:300, ab150129, Abcam).

### RNA Isolation and Real-Time Quantitative PCR

Total RNA was isolated from purified primary cells or tissues by using total RNA extraction kit, and reverse transcription was performed using the ReverTra Ace^®^ qPCR RT Kit according to the manufacturer’s instructions. RNA concentration and quality were assessed using a NanoDrop instrument (Isogen Life Science, Belgium). PCR reactions were carried out using SYBR Green^®^ Realtime PCR Master Mix and the amplification was performed with a LightCycler^®^ 480 System (Roche Molecular Systems, Inc., CA). PCR primers were designed using Primer-BLAST from the National Center for Biotechnology Information. The following primers were used: IL-6, forward, 5′-TAGTCCTTCCTACCCCAACTTCC-3′; IL-6, reverse, 5′-TTGGTCCTTAGCCACTCCTTC-3′; IL-1β, forward 5′-AGCTTTCGACAGTGAGGAGAATGA-3′; IL-1β, reverse, 5′- AAGCTCTTGTCGAGATGCTGC-3′; iNOS, forward, 5′-CAAC AACGTGGAGAAAACCCC-3′; iNOS, reverse, 5′-AGGGATT CTGGAACATTCTGTG-3′; MIP-1α, forward 5′-GCTTCTCC TATGGACGGCAA-3′; MIP-1α, reverse, 5′-GGTCAGGAAAA TGACACCCG-3′; TNFα, forward, 5′-ATGGGCTCCCTCTCAT CAGT-3′; TNFα, reverse, 5′-GCTTGGTGGTTTGCTACGAC-3′. The PCR products were verified by electrophoresis on a 2% agarose gel containing ethidium bromide.

### Partial Sciatic Nerve Ligation (PSNL)

A total sixty SD rats were used, and the group information is shown in the method part of **BoNT/A Administration**. Partial ligation of the sciatic nerve was performed using a previously reported method (Malmberg and Basbaum, 1998). In general, rats were first anesthetized with pentobarbital (50 mg/kg) and fixed on a surgery table. And then, a small incision was made in the right hind limb to expose the sciatic nerve. Partial sciatic nerve injury was produced by tying a tight ligature with a 9–0 silk suture around approximately half the diameter of the sciatic nerve. Finally, skin was closed, and the wound was applied with iodine for protecting from infection. For control rats (sham-operated), the nerve was exposed without ligation.

### BoNT/A Administration

BoNT/A was dissolved in 0.9% saline at a concentration of 200 U/mL as a stock solution stored in liquid nitrogen. The stock solution was diluted prior to administration. For *in vivo* experiments, sixty rats were divided into ten groups (*n* = 6 per group): (1) Control (nerve exposed without ligation), (2) Saline *i.t.* pre D2 + PSNL (saline *i.t.* administrated to rats 2 days before PSNL surgery), (3) PSNL + Saline *i.pl.* post D3 (saline *i.pl.* administrated to rats 3 days after PSNL surgery), (4) BoNT/A (20 U/kg *i.pl.* administrated to rats without injury), (5 and 6) PSNL + BoNT/A *i.pl.* post D3 (BoNT/A 10 U/kg or 20 U/kg *i.pl.* administrated to rats 3 days after PSNL surgery), (7) PSNL + BoNT/A *i.t.* post D3 (BoNT/A 1 U/kg *i.t.* administrated to rats 3 days after PSNL surgery), (8 and 9) BoNT/A *i.t.* pre D2 + PSNL (BoNT/A 0.5 U/kg or 1 U/kg *i.t.* administrated to rats 2 days before PSNL surgery) groups, (10) BoNT/A *i.pl.* pre D2 + PSNL (BoNT/A 10 U/kg administrated to rats 2 days before PSNL surgery) groups. BoNT/A was administrated into rats according to a reported method (Hylden and Wilcox, 1980). Briefly, under the anesthetization with pentobarbital (50 mg/kg), injections were given by using a Hamilton microsyringe connected to a tube with a 30-gage hypodermic needle. For *i.pl.* administration, 200 U/mL BoNT/A solution (10 and 20 U/kg BoNT/A, that is 12.5 and 25 μL of BoNT/A solution each paw) was injected into the plantar surface of the injury side of rats’ hind paw (right hind paw) 2 days before or 3 days after PSNL injury via a hypodermic needle. For *i.t.* administration, 200 U/mL BoNT/A solution (0.5 or 1 U/kg BoNT/A, that is 0.6 or 1.5 μL of BoNT/A solution each paw) was intrathecally injected into the space between the L5 and L6 spinal segments of rats 2 days before or 3 days after PSNL injury. The dose of BoNT/A application were chosen with no toxic units on the basis of previous report (Cui et al., 2004) and our maximal dose of BoNT/A (20 U/kg, *i.pl.*) were used in similar animal studies from Favre-Guilmard et al. (2009) (*i.pl.*, 20 U/kg in rats), Xiao et al. (2011) (*i.pl.*, 10 U and 20 U/kg in rats), Gui et al. (2020) (*i.pl.*, 10 U and 20 U/kg in rats) and Park et al. (2006)(*i.pl.*, 10–40 U/kg in rats). 100 U are equivalent to approximate 4.8 ng of BoNT/A as reported by Cui et al. (2004).

For the *in vitro* experiments, BoNT/A was diluted into a culture medium at the concentration of 2 U/mL for astrocytes and 1 U/mL for microglia. Astrocytes or microglia were cultured in a PLL-coated 6-well plate at a density of 3 × 10^4^. After purification, the culture medium was replaced by DMEM (10% FBS) containing BoNT/A. Forty-eight hours later, the medium was removed and the cultured cells were gently washed with pre-warmed DMEM twice, and then replaced by DMEM (10% FBS) containing LPS (100 ng/mL for astrocytes and 10 ng/mL for microglia). Twenty four or forty eight hours later, morphological changes and the mRNA levels of inflammatory cytokines were detected. We use lower concentration of BoNT/A for microglia pretreatment compared with that for astrocyte based on the ultra-sensitivity of microglia to environmental changes (Tan et al., 2020). The concentration of BoNT/A used for cultured cells were chosen with no toxic units on the basis of a previous report (Piotrowska et al., 2017). We should note that the dosage of BoNT/A used *in vivo* experiments appears much lower compared to those *in vitro* ones. Considering their high degree of toxicity for the organism, we used high concentration of BoNT/A (200 U/mL) with a small volume amount during *in vivo* experiment, so that the toxin will not diffuse locally or spread far from the original injection site. Since the BoNT/A was locally administrated *in vivo*, the final effective concentration of BoNT/A could not such differ from that *in vitro* condition.

### Behavioral Test

Mechanical sensitivity was assessed by the mechanical paw pressure test, as previously described (Matsumoto et al., 2006; Xie et al., 2010). Briefly, rats were placed in Plexiglas chambers on a 6 × 6 mm wire mesh grid floor and were allowed to acclimate for a period of 1 h. A mechanical stimulus was then delivered to the right hind paw using an automated transducer indicator (ALMEMO@ 2450, IITC Inc., Woodland Hills, United States). The amount of pressure that induced a flexor response was defined as the pain threshold. A cutoff pressure of 40 g was set to avoid tissue damage. Thermal sensitivity was assessed by the Hargreaves thermal paw withdrawal test as previously described (Hargreaves et al., 1988; Xie et al., 2010). Briefly, rats were placed in Plexiglas chambers on top of a glass sheet and were allowed to acclimate for a period of 1 h. A thermal stimulator (IITC Inc., Woodland Hills, CA, United States) was positioned under the glass sheet and the focus of the projection bulb was precisely aimed at the middle of the plantar surface of the animal. A mirror attached to the stimulator permitted visualization of the plantar surface. A cutoff latency of 30 s was set to avoid tissue damage. In the mechanical and thermal tests, the thresholds and latencies were determined as the averages of three repeated challenges at 10 min intervals. Observers scoring the responses were blinded to the given pretreatment.

### Statistical Analysis

Three or more independent measurements of tested parameters were performed. Data were presented as the mean ± SEM. Statistical analysis was performed with Origin Pro8 (RRID: SCR_014212; OriginLab, Haverhill, MA, United States). Significant changes were identified using A non-parametric two-tailed *t*-test (Mann Whitney test), at 95% confidence interval with *p* < 0.05, or non-parametric Kruskal-Wallis test with *p* < 0.05 considered as statistically significant (*p*-values: ^∗^ < 0.05, ^∗∗^ < 0.01).

## Results

### BoNT/A Pretreatment Inhibits Lipopolysaccharide (Lipopolysaccharide)-Induced Microglia Activation

**FIGURE 1:**
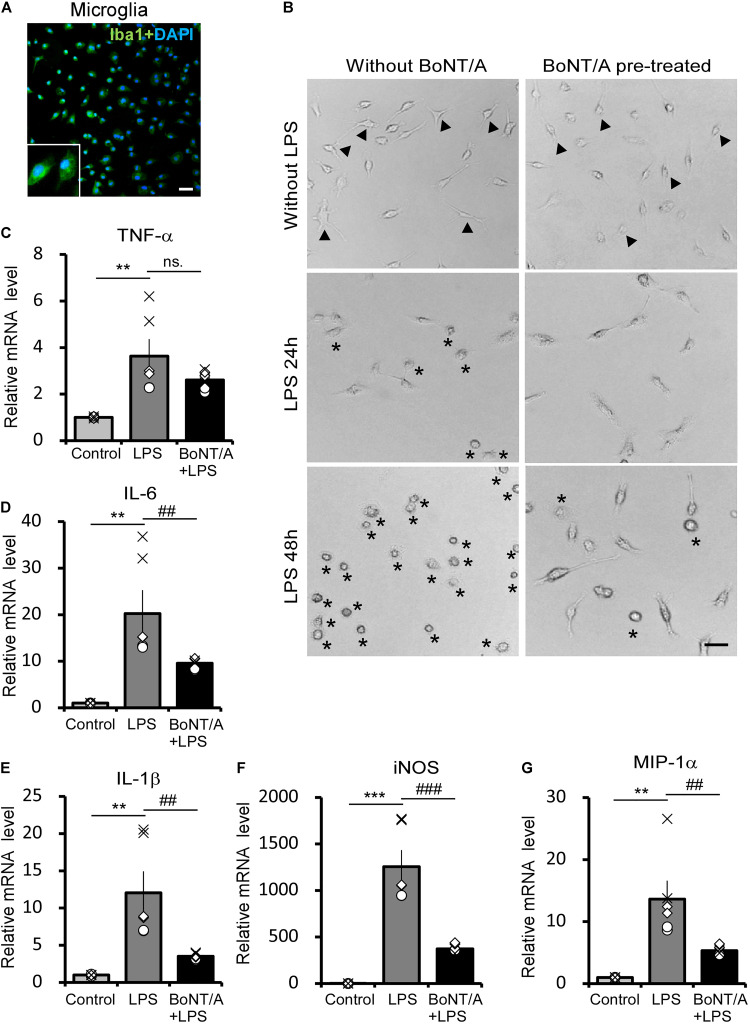
Botulinum neurotoxin A (BoNT/A) pretreatment inhibits lipopolysaccharide (LPS)-activated morphological changes and inflammatory cytokine release in primary microglia. **(A)** Image showing double-staining with Iba1 (a specific marker of microglia, green) and 4′,6-diamidino-2-phenylindole (DAPI, blue) in purified microglia from the cerebral cortices of newborn Sprague-Dawley (SD) rats. Scale bar: 100 μm. **(B)** Representative morphologies of cultured microglia visualized with a phase contrast microscope. Cells were pretreated with or without BoNT/A (1 U/mL) for 48 h prior to incubation with LPS (10 ng/mL) for 24 or 48 h. Arrowheads indicate the representative resting microglia, showing either a typical elongated, bipolar, or unipolar shape. Asterisks indicate the representative activated microglia which exhibit a typically ameboid morphology. Scale bar: 50 μm. **(C–G)** mRNA expression of tumor necrosis factor alpha (TNF-α, **C**), interleukin-6 (IL-6, **D**), interleukin-1β (IL-1β, **E**), inducible nitric oxide synthase (iNOS, **F**), and macrophage inflammatory protein (MIP)-1α **(G)** relative to β-actin. Data are presented as the fold change compared to control group (without any treatment). BoNT/A pretreatment significantly reduced the LPS-induced upregulation of the mRNA levels of TNF-α, IL-6, IL-1β, iNOS, and MIP-1α. Three independent experiments were performed with 2 replicates for each experiment. Data are represented as the mean ± SEM. A non-parametric two-tailed *t*-test was used for comparison. ***p < 0.001, **p < 0.01 compared to control group; ^###^*p* < 0.001, ^##^*p* < 0.01, and ns. denotes no significance compared to the LPS-treated group.

To identify the interaction and function of BoNT/A on microglia, we examined LPS-activated morphological changes and inflammatory cytokine release in primary microglia with or without BoNT/A pretreatment. Microglia were isolated and purified from neonatal rats, and the purity was confirmed by immunostaining for Iba1 (>95%), a marker of microglia (Figure 1A). Twelve hours after purification, microglias were treated with or without BoNT/A (1 U/mL) for 48 h. Medium with free BoNT/A were removed before the application of new media containing LPS (10 ng/mL). Microglia without any treatment displayed a resting state similar to that under physiological condition, which is characterized by several processes extending from a thin cell body. Without BoNT/A pretreatment, LPS incubation resulted in the gradual transformation of resting microglia into an activated state characterized by an ameboid form, while BoNT/A-pretreated microglia displayed a more ramified cell type (Figure 1B), suggesting that the pretreatment of BoNT/A inhibited the LPS-induced morphological transformation of microglia and, therefore, the transition from an inactivated to activated cell type.

In addition to morphological observation, total mRNA was extracted from these cells and the mRNA levels of inflammatory cytokines and chemokines were characterized. Without BoNT/A pretreatment, LPS incubation induced an approximately 3. 5-, 20-, and 12-fold increase in the mRNA levels of the pro-inflammatory cytokines tumor necrosis factor alpha (TNF-α, Figure 1C), interleukin-6 (IL-6, Figure 1D), and interleukin-1β (IL-1β, Figure 1E), respectively. However, the expression of pro-inflammatory cytokines IL-6 and IL-1β was significantly decreased to approximately 47 and 29% in the BoNT/A pretreated microglia compared with the BoNT/A untreated ones in response to LPS stimulation, while TNF-α expression has no clear difference between the two group (Figures 1C–E). Moreover, LPS induced a substantial increase in the mRNA levels of inducible nitric oxide synthase (iNOS, ∼1,200-fold increase, Figure 1F) and macrophage inflammatory protein (MIP)-1α (∼14-fold increase, Figure 1G) in microglia, while the extent of increase was significantly lower in BoNT/A pretreated microglia (39 and 30%, respectively, of the expression level in BoNT/A untreated microglia, Figures 1F,G). From these results, we conclude that pretreatment of BoNT/A could interact with microglia which further inhibits LPS-induced microglia activation and pro-inflammatory cytokine release.

### BoNT/A Pretreatment Does Not Affect Lipopolysaccharide -Induced Inflammatory Cytokines Release in Primary Astrocytes

**FIGURE 2:**
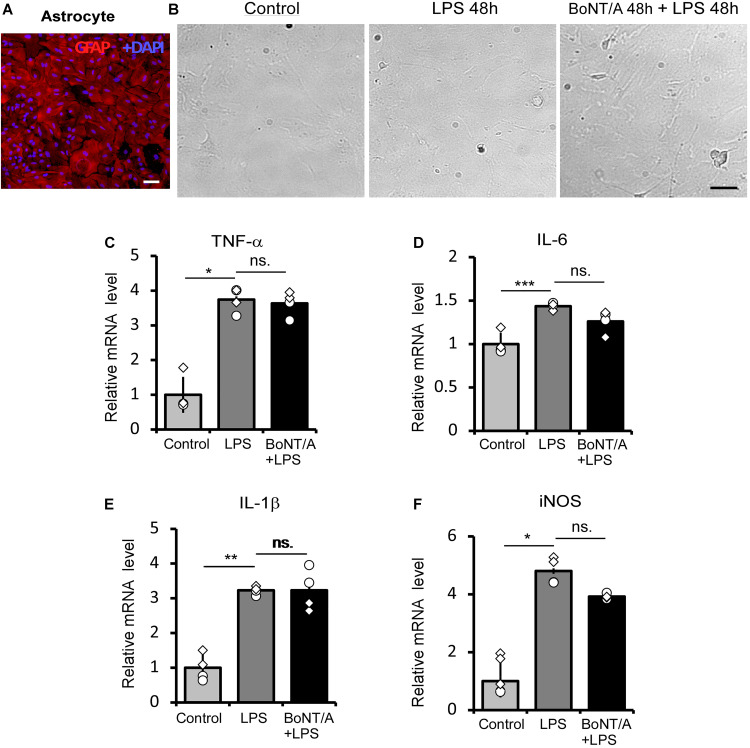
BoNT/A pretreatment does not affect LPS-induced inflammatory cytokine release in primary astrocytes. **(A)** Image showing double staining with glial fibrillary acidic protein (GFAP, a specific marker of astrocytes, red) and DAPI (blue) in purified astrocytes from the cerebral cortices of newborn SD rats. Scale bar: 200 μm. **(B)** Representative morphologies of cultured astrocytes visualized with a phase contrast microscope. Cells were pretreated with or without BoNT/A (2 U/mL) for 48 h prior to incubation with LPS (100 ng/mL) for 48 h. The morphologies of astrocytes with or without LPS incubation show no clear differences. Scale bar: 200 μm. **(C–F)** mRNA expression levels of TNF-α **(C)**, IL-6 **(D)**, IL-1β **(E)**, and iNOS **(F)** relative to β-actin. BoNT/A pretreatment did not affect the LPS-induced upregulation of mRNA levels of TNF-α, IL-6, IL-1β, and iNOS. Data are presented as the fold change compared to the control group (without any treatment). Three independent experiments were performed with 2 replicates for each experiment. Data are represented as the mean ± SEM. A non-parametric two-tailed *t*-test was used for comparison. ***p < 0.001, **p < 0.01, and *p < 0.05 compared to the control group, ns. = no significance.

We next checked the influence of BoNT/A pretreatment on LPS-treated astrocytes. Astrocytes were purified from neonatal rats and the purity (>95%) was confirmed by immunostaining for the astrocyte marker glial fibrillary acidic protein (GFAP) (Figure 2A). The purified astrocytes were treated with or without BoNT/A (2 U/mL) for 48 h, free BoNT/A was removed, and cells were treated with LPS (100 ng/mL) for another 48 h. Under a microscope, we did not observe any morphological change when applying high concentrations of LPS to astrocytes, regardless of whether they were pretreated with BoNT/A or not (Figure 2B). LPS treatment significantly increased the mRNA expressions of TNF-α, IL-6, IL-1β, and iNOS, although there is no clear difference in the expression levels of these cytokines and chemokines differences with or without BoNT/A pretreatment (Figures 2C–F). These results indicate that pretreatment with BoNT/A does not affect LPS-induced astrocyte activation.

### BoNT/A Intrathecal Pre-administration Partially Prohibits Partial Sciatic Nerve Ligation -Induced Pro-inflammatory Cytokines in Rats

**FIGURE 3:**
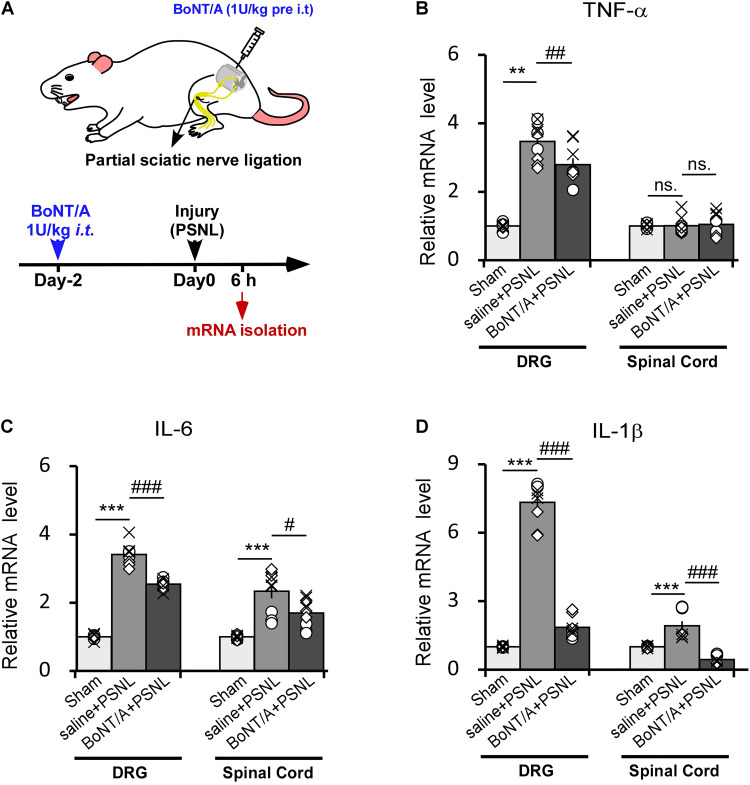
Intrathecal pre-administration of BoNT/A partially prohibits partial sciatic nerve ligation (PSNL)-induced pro-inflammatory cytokine release in rat DRGs and the spinal cord dorsal horn. **(A)** A schematic illustration showing the protocol where BoNT/A was intrathecally (*i.t.*, 1 U/kg) injected into spaces between the L5 and L6 spinal segments 2 days before PSNL surgery in rats. Six hours after PSNL surgery, the total mRNA was extracted from the DRGs and ipsilateral spinal cord dorsal horn, and the mRNA expression levels of the pro-inflammatory cytokines were detected. **(B–D)** mRNA expression levels of TNF-α **(B)**, IL-6 **(C)**, and IL-1β **(D)** relative to β-actin. Data are presented as the fold change compared to the control group (nerve was exposed without ligation). Three mice were used for each group with 4 replicates for each experiment. Data are represented as the mean ± SEM. A non-parametric two-tailed *t*-test was used for comparison. ***p < 0.001 and **p < 0.01 compared to control group, ^###^*p* < 0.001, ^##^*p* < 0.01, and ^#^*p* < 0.05 compared to the PSNL group. ns. = no significance.

We next injected BoNT/A into spaces between the L5 and L6 spinal segments of rats to inhibit the initial activation of microglia. 2 days later, the rats were subjected to PSNL surgery. 6 h after PSNL surgery, mRNAs from the dorsal root ganglia (DRG) and spinal cord dorsal horn were isolated and analyzed by qPCR (Figure 3A). In ipsilateral DRGs, PSNL surgery led to a significant upregulation of the mRNA levels of TNF-α, IL-6 and IL-1β compared with the control group (sham-operated, the nerve was exposed without ligation as described in method section, Figures 3B–D), which is in line with previous report (Martin et al., 2019). A single intrathecal (*i.t.*) pre-administration of a low dose BoNT/A (1 U/kg) significantly diminished the PSNL-induced upregulation of the mRNA levels of TNF-α (Figure 3B, 2.8 ± 0.2-fold change for BoNT/A vs. 3.6 ± 0.2-fold change for saline *i.t.* pre-administration), IL-6 (Figure 3C, 2.6 ± 0.2-fold change for BoNT/A vs. 4.2 ± 0.9-fold change for saline *i.t.* pre-administration) and IL-1β (Figure 3D, 1.7 ± 0.3-fold change for BoNT/A vs. 7.4 ± 0.3-fold change for saline i.t. pre-administration). In the ipsilateral spinal cord dorsal horn, the mRNA levels of IL-6 and IL-1β were significantly upregulated after PSNL injury (Figures 3C,D), while that of TNF-α was not (Figure 3B). Compared with the control group, *i.t.* pre-administration of BoNT/A significantly diminished the PSNL-induced upregulation of IL-1β (Figure 3D, 1.1 ± 0.3-fold change for BoNT/A *i.t.* administration before PSNL and 3.0 ± 0.2 fold change for saline *i.t.* administration before PSNL), whereas the regulation of IL-6 was not affected (Figure 3C). These results indicate that a single *i.t.* pre-administration of BoNT/A partially inhibits the PSNL-induced upregulation of pro-inflammatory cytokines not only in spinal cord, but also in DRGs in rats.

### Intrathecal Pre-administration of a Low Dose of BoNT/A Efficiently Prevents Partial Sciatic Nerve Ligation -Induced Neuropathic Pain-Like Behaviors in Rats

**FIGURE 4:**
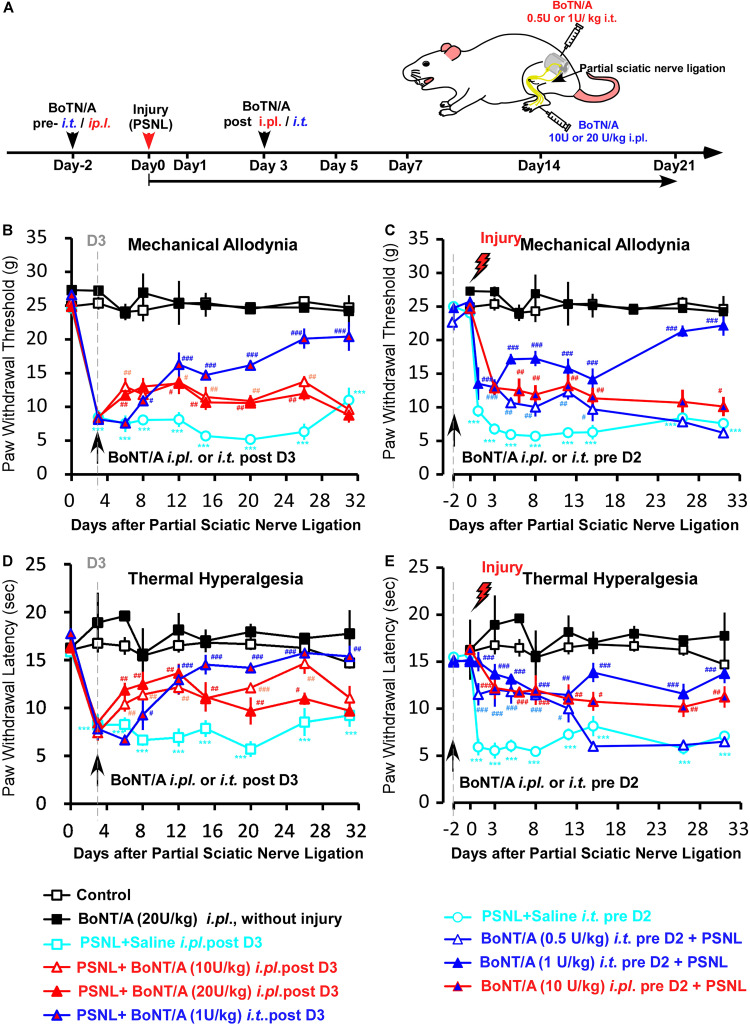
A low dose of BoNT/A intrathecal (*i.t*) pre-administration prevents PSNL-induced mechanical allodynia and thermal hyperalgesia in rats. **(A)** A schematic illustration of the experimental protocol. Botulinum toxin A (BoNT/A) was intrathecally (*i.t*) injected into spaces between the L5 and L6 spinal segments) 2 days before surgery or into the plantar surface of the paw (*i.pl.*) 3 days after the PSNL surgery in rats. Mechanical allodynia and thermal hyperalgesia behavior tests were performed after PSNL surgery. Mechanical allodynia **(B,C)** and thermal hyperalgesia **(D,E)** were compared between the control (nerve exposed without ligation), Saline *i.t.* pre D2 + PSNL (saline *i.t.* administrated to rats 2 days before PSNL surgery), PSNL + Saline *i.pl.* post D3 (saline *i.pl.* administrated to rats 3 days after PSNL surgery), BoNT/A (20 U/kg *i.pl.* administrated to rats without injury), PSNL + BoNT/A *i.pl.* post D3 (BoNT/A 10 U/kg or 20 U/kg *i.pl.* administrated to rats 3 days after PSNL surgery), PSNL + BoNT/A *i.t.* post D3 (BoNT/A 1 U/kg *i.t.* administrated to rats 3 days after PSNL surgery), BoNT/A *i.t.* pre D2 + PSNL (BoNT/A 0.5 U/kg or 1 U/kg *i.t.* administrated to rats 2 days before PSNL surgery) and BoNT/A *i.pl.* pre D2 + PSNL (BoNT/A 10 U/kg administrated to rats 2 days before PSNL surgery) groups. Statistical analysis revealed that the paw withdrawal threshold and paw withdrawal latency were significantly higher after BoNT/A application. Six mice were used in each group. Data are represented as the mean ± SEM. Non-parametric Kruskal-Wallis test was used for comparison. ***p < 0.001 compared to the sham group; ^###^*p* < 0.001, ^##^*p* < 0.01, and ^#^*p* < 0.05 compared to the PSNL group.

Given that the production of pro-inflammatory cytokines and chemokines is a critical step in driving peripheral and central sensitization after peripheral nerve injury, we examined the effect of BoNT/A pretreatment on PSNL-induced neuropathic pain-like behaviors in rats and compared routes of administration of BoNT/A in preventing PSNL-induced mechanical allodynia and thermal hyperalgesia. Rats were subjected to either a single *i.t.* (0.5 or 1 U/kg) or a single *i.pl.* (10 or 20 U/kg) injection of BoNT/A 2 days before or 3 days after PSNL surgery (Figure 4A). Mechanical sensitivity was assessed by the mechanical paw pressure test (Figures 4B,C) and thermal sensitivity was assessed using the Hargreaves thermal paw withdrawal test (Figures 4D,E). The maximal dose of BoNT/A with no toxic units was used in similar animal studies from previous reports (Park et al., 2006; Favre-Guilmard et al., 2009; Xiao et al., 2011; Gui et al., 2020), and no obvious change in general health factors such as body weight or breathing rhythm were observed after BoNT/A administration and motor functions also appears normal in these rats which is in line with previous report (Park et al., 2006). The paw withdrawal thresholds of the rats subjected to PSNL surgery were significantly decreased after injury, and strong mechanical allodynia persisted for 31 days (Figures 4B,C). A single *i.pl.* injection of BoNT/A (2 days before or 3 days after the operation) relieved PSNL-induced mechanical allodynia to a limited extent (Figure 4B). Interestingly, a single *i.t.* injection of a low dose BoNT/A (1 U/kg) 2 days before the operation significantly prevented PSNL-induced mechanical allodynia immediately after PSNL surgery and this difference was maintained for at least 31 days, while the *i.t.* post- administration of BoNT/A requires longer time to influence pain relief compared with the other three types of BoNT/A administrations (*ip.l.* pre-, *i.t* pre-. and *i.pl.* post-) (Figures 4B–E).

Similar to the result of the mechanical paw pressure test, PSNL surgery resulted in a significantly reduced thermal threshold that persisted for at least 31 days, and a single *i.pl.* injection of BoNT/A (2 days before or 3 days after the operation) significantly inhibited PSNL-induced thermal hyperalgesia (Figures 4D,E). Importantly, a single *i.t.* injection of a low dose of BoNT/A (1 U/kg) 2 days before the operation efficiently prevented PSNL-induced thermal hyperalgesia (Figure 4E). Taken together, *i.t.* pre-administration of a low dose BoNT/A efficiently prevented PSNL-induced mechanical allodynia and thermal hyperalgesia in rats. And *i.t.* pre-injection of BoNT/A was more effective than *i.pl.* pre- or post- injection of BoNT/A in preventing both mechanical allodynia and thermal hyperalgesia, even though the dose was 20 times lower. Combining with the *in vitro* results, we suggest that efficiency of *i.t.* pre-injection of BoNT/A could result from the effective inhibition of spinal microglia activation induced by PSNL injury.

## Discussion

In this study, we found that BoNT/A pretreatment significantly inhibits LPS-induced activation and pro-inflammatory cytokine release in primary microglia when free BoNT/A was removed while there was no effect on the activation of astrocytes. We suggest that BoNT/A could directly interact with microglia prior to LPS application, which further affects the initial LPS-induced microglia activation. In agreement with the *in vitro* result, a single *i.t.* administration of low dose BoNT/A before PSNL surgery significantly prohibited PSNL-induced upregulation of pro-inflammatory cytokines in the spinal cord dorsal horn and DRGs, resulting in PSNL-induced neuropathic pain-like behaviors being effectively prevented. We conclude that BoNT/A can directly interact with microglia prior to the stimulation of inflammation which thus inhibits the initial microglia activation, and in turn prevents the PSNL-induced neuropathic pain.

The effect of BoNT/A on glial cells is widely studied during the past decade, however, whether BoNT/A could have a direct interaction with microglia or astrocytes is still in debate. Previous report demonstrated that BoNTs can enter not only neurons but also non-neuron cells such as astrocyte via endocytic processes (Verderio et al., 1999). And Marinelli et al. (2012) found that the cleavage of BoNT/A substrate SNAP-25 (clSNAP-25) was detectable in CCI-activated spinal astrocytes but not in microglia after *i.pl.* administration of BoNT/A in mice, suggesting a direct action of BoNT/A on astrocytes. In contrast, Matak et al. (2012) were unable to detect the clSNAP-25 proteins in spinal astrocyte treated with BoNT/A in naïve rats. By *in vitro* studies, Piotrowska et al. (2017) suggested that BoNT/A could have a direct action on primary microglia rather than astrocyte on inhibition of the LPS-induced glial cell activation. In our study, we used the concentration of BoNT/A with no toxic units on cultured cells as Piotrowska et al. (2017) reported with a different way of BoNT/A pre-treatment. Instead of letting the BoNT/A remain in the medium containing LPS, we removed the BoNT/A in order to mimic the *in vivo* condition that BoNT/A could be taken in by the surrounding neurons as soon as it was administrated. And we found that BoNT/A pretreatment, even under the condition that BoNT/A was removed prior to LPS stimulation, did not affect the activation of astrocytes (Figure 2), while significantly inhibited LPS-induced activation and pro-inflammatory factor release in cultured microglia (Figure 1). Our results are in line with the suggestion of Piotrowska et al. (2017), and additionally support the idea that BoNT/A could have a direct molecular target on microglia but not on astrocyte.

In our study, we did not examine the detail ways with which BoNT/A could interact on microglia. We suggest that BoNT/A could probably directly bind to some protein receptors in microglia and blocks their activity in response to LPS, resulting in the failure of LPS-induced microglia activation even though free BoNT/A had been removed from the medium. Another possibility is that BoNT/A may enter microglia via unknown receptors which interfere with microglia activation-related protein expression and release. In neural cells, apart from the well-known BoNT/A -binding molecule SV2, an increasing number of studies have suggested that BoNT/A could enter cells via binding to some other molecules, such as fibroblast growth factor receptor 3 (FGF3) (Jacky et al., 2013) and gangliosides (Rummel et al., 2004). Besides this, BoNT/A has been reported to enter neural cytosol by interaction with transient receptor potential vanilloid 1 (TRPV1) (Li and Coffield, 2016). However, there is currently no evidence showing any membrane acceptor of BoNT/A in microglia, although there are several *in vitro* studies suggesting that BoNT/A could possibly target non-neuronal cells (Zhibo and Miaobo, 2008; Oh et al., 2012; da Silva et al., 2015). Further investigations are required to characterize the acceptors in microglia in greater detail.

Currently, the effect of BoNT/A on inflammation is still greatly debated. Cui et al. (2004) found that the *i.pl.* injection of BoNT/A reduced formalin-induced peripheral sensitization in rats. Mika and coworkers showed that the *i.pl.* injection of BoNT/A reduced the chronic constriction injury (CCI)-induced microglia activation (Mika et al., 2011; Vacca et al., 2013) and pro-inflammatory cytokine release in rats (Zychowska et al., 2016). However, Bach-Rojecky et al. (2008) demonstrated that the *i.pl.* pre-injection of BoNT/A has no anti-inflammatory action in carrageenan- and capsaicin-induced inflammation in rats, although pain hypersensitivity was reduced. These contradictory results could contribute to the difference between different injury models. Our study used a rat PSNL model, and we found that TNF-α, IL-6, and IL-1β are highly upregulated in response to PSNL injury in DRGs (Figure 3). These pro-inflammatory cytokines are mainly produced by immune cells and satellite glial cells in DRGs. Upregulation of these cytokines could activate a mosaic of peripheral receptors, resulting in peripheral sensitization, such as through TNF-α (Jin and Gereau, 2006) and IL-1β (Liu et al., 2016) which could initiate peripheral sensitization by increasing TRPV1 and NaV1.8 activity, respectively. We found that *i.t.* pre-administration of BoNT/A significantly prohibited the upregulation of these factors in DRG, which further prohibited PSNL-induced peripheral sensitization. In addition to the response in DRGs, IL-6 and IL-1β were also rapidly upregulated in the spinal cord dorsal horn following PSNL injury (Figure 3), and *i.t.* pre-administration of BoNT/A significantly prohibited IL-1β upregulation in the spinal cord dorsal horn. IL-6 and IL-1β are important neuromodulators for inducing central sensitization, and they are mainly produced by microglia in an early stage in response to injury (i.e., 6 h after PSNL) because the activation of spinal astrocytes usually occurs about 4 days after microglia activation (Scholz and Woolf, 2007; Romero-Sandoval et al., 2008). IL-1β is reported to enhance N-methyl-D-aspartate (NMDA) receptor-mediated intracellular calcium increase via activation of tyrosine protein kinase Src (Viviani et al., 2003), and IL-1β is also reported to inhibit GABA_A_ receptor-mediated currents in cultured hippocampal neurons (Wang et al., 2000). We thus suggest that *i.t.* pre-administration of BoNT/A prohibit PSNL-induced peripheral and central sensitization in rats.

The anti-nociceptive effect of BoNT/A was widely investigated *in vivo* in rodents. However, to our knowledge, BoNT/A’s effect on glial cells was demonstrated only in CCI-induced neuropathic pain in mice and rats (Luvisetto et al., 2007; Marinelli et al., 2010; Mika et al., 2011). Our results indicate that BoNT/A are also able to inhibit microglia activation in PSNL-induced neuropathic pain in rats which could suggesting potential different populations of human neuropathic pain patients. Both a previous report (Piotrowska et al., 2017) and our results indicated that BoNT/A can directly act on microglia, thus *i.t.* administration of BoNT/A could be more effective to inhibit microglia activation resulting in a faster and more long-lasting analgesic effect compared to local *i.pl.* in chronic pain. And our results indeed demonstrated that a single i.t. pre-administration was more effective in reduction of mechanical and thermal hypersensitivity than i.pl. injection (Figure 4). The anti-nociceptive effects of BoNT/A *i.t.* administration effects was also demonstrated in diabetic neuropathy (Bach-Rojecky et al., 2010), CCI- induced neuropathic pain (Marinelli et al., 2010), carrageenan-induced mirror pain (Drinovac Vlah et al., 2016) and acidic saline-induced pain (Bach-Rojecky and Lacković, 2009). Among these studies, higher efficiency in i.t. administration compared with i.pl. injection was reported in diabetic neuropathy (Bach-Rojecky et al., 2010) and CCI -induced neuropathic pain (Marinelli et al., 2010). We suggest that it could result from the direct inhibition of BoNT/A on the spinal microglia activation during central sensation during chronic pain processes. On the other hand, *i.t.* administration of BoNT/A could inhibit neuronal hyper-sensitization via the excitatory neurotransmission blockage in the spinal cord. Further investigations are required to clarify the underlying mechanisms in greater detail.

## Conclusion

In conclusion, we demonstrated that BoNT/A pretreatment directly inhibit LPS-induced activation and pro-inflammatory cytokine release in primary microglia and intrathecal pre-administration of BoNT/A significantly prevented PSNL injury- induced acute pain and the further chronic pain. Our results might contribute to better understanding of the analgesic effect of BoNT/A in chronic pain and further development the use of BoNT/A in microglia hyperactivation- caused disease.

## Data Availability Statement

The raw data supporting the conclusions of this article will be made available by the authors, without undue reservation.

## Ethics Statement

The animal study was reviewed and approved by the Shenzhen Institutes of Advanced Technology, Chinese Academy of Sciences Research Committee of Laboratory Animals.

## Author Contributions

LX, WX, and YQ initiated and designed the research. XF, DX, JL, and WX performed the experiments. XF, YQ, and WX wrote the manuscript. All authors have read and agreed to the published version of the manuscript.

## Conflict of Interest

The authors declare that the research was conducted in the absence of any commercial or financial relationships that could be construed as a potential conflict of interest.

## Publisher’s Note

All claims expressed in this article are solely those of the authors and do not necessarily represent those of their affiliated organizations, or those of the publisher, the editors and the reviewers. Any product that may be evaluated in this article, or claim that may be made by its manufacturer, is not guaranteed or endorsed by the publisher.
